# *Poria cocos* (Schw.) Wolf, a Traditional Chinese Edible Medicinal Herb, Promotes Neuronal Differentiation, and the Morphological Maturation of Newborn Neurons in Neural Stem/Progenitor Cells

**DOI:** 10.3390/molecules28227480

**Published:** 2023-11-08

**Authors:** Xia Jiang, Zhaotun Hu, Xiaoyan Qiu, Liming Wu, Rong Zhou, Yaoyao Yang, Xiaoliang Xiang

**Affiliations:** 1 Key Laboratory of Research and Utilization of Ethnomedicinal Plant Resources of Hunan Province, College of Biological and Food Engineering, Huaihua University, Huaihua 418008, China; jiangxia285333006@163.com (X.J.); huzhaotun@hhtc.edu.cn (Z.H.); qxy199002@163.com (X.Q.); stannio@163.com (L.W.); suduzhongyao1234@163.com (R.Z.); 15576594742@163.com (Y.Y.); 2 College of Chemistry and Materials Engineering, Huaihua University, Huaihua 418008, China; 3 Hunan Provincial Higher Education Key Laboratory of Intensive Processing Research on Mountain Ecological Food, Huaihua University, Huaihua 418008, China

**Keywords:** *Poria cocos* (Schw.) Wolf, edible medicinal herb, neural progenitor cells, neurogenesis, neuronal differentiation

## Abstract

Neurogenesis in the adult brain comprises the entire set of events of neuronal development. It begins with the division of precursor cells to form a mature, integrated, and functioning neuronal network. Adult neurogenesis is believed to play an important role in animals’ cognitive abilities, including learning and memory. In the present study, significant neuronal differentiation-promoting activity of 80% (*v*/*v*) ethanol extract of *P. cocos* (EEPC) was found in Neuro-2a cells and mouse cortical neural stem/progenitor cells (NSPCs). Subsequently, a total of 97 compounds in EEPC were identified by UHPLC-Q-Exactive-MS/MS. Among them, four major compounds—Adenosine; Choline; Ethyl palmitoleate; and L-(-)-arabinitol—were further studied for their neuronal differentiation-promoting activity. Of which, choline has the most significant neuronal differentiation-promoting activity, indicating that choline, as the main bioactive compound in *P. cocos*, may have a positive effect on learning and memory functions. Compared with similar research literature, this is the first time that the neuronal differentiation-promoting effects of *P. cocos* extract have been studied.

## 1. Introduction

Neurogenesis and subsequent neurite outgrowth are critical processes for the formation of functional neural networks during brain development [1]. In neurodegenerative diseases such as Alzheimer’s disease (AD) or Parkinson’s disease (PD), extensive neuronal loss and neurite atrophy are the major characteristics during the pathogenesis of the disease [2]. Furthermore, Neurite loss is one of the typical features of neuronal injury. Neural stem/progenitor cells (NSPCs) are multi-potent cells that are able to self-renew and differentiate into neurons [3]. Therefore, promoting neuronal differentiation is an effective strategy for stem cell therapy and neural regeneration, which contributes to the reconstruction of neural circuits after neurodegeneration and brain injury [4]. So far, a variety of natural products and their derivatives have been found to have neuronal differentiation-promoting activity and have been studied for neural regeneration.

*Poria cocos* (Schw.) Wolf (*P. cocos*) is a fungus used as a traditional Chinese edible medicinal herb [5]. *P. cocos* is applied in 10% of clinical Chinese medicine prescriptions and is known as one of the four major raw materials in China [6]. According to the Chinese Pharmacopoeia, *P. cocos* has the effect of promoting diuresis, eliminating dampness, strengthening the spleen, and calming the heart [7]. It is clinically used in traditional Chinese medicine to treat memory loss caused by spleen deficiency, abnormal transportation and transformation, and phlegm obstruction. In addition, the chemical constituents in *P. cocos* were widely used in clinical treatments due to their antioxidant, anti-tumor, anti-inflammatory, anti-bacterial, anti-aging, and other effects [5].

Current studies have found that some traditional Chinese medicine prescriptions with *P. cocos* as the main raw material have potential effects on the treatment of neurodegenerative diseases. For instance, Bu-wang-san is a classical traditional Chinese medicinal formula for enhancing memory. It has been reported that BWS could improve learning and memory functions through its effect on synaptic plasticity in ovariectomized rats [8]. Another traditional Chinese medicinal formula, Kaixin powder, is reported to alleviate chronic stress-induced depressive symptoms by upregulating the expression of synaptic proteins in the rat hippocampus [9]. Traditionally, Liu-wei-di-huang decoction is considered to have a beneficial neuro-protect effect on the cognitive deficits of diabetic encephalopathy [10].

Also, Shen-hui soup is widely used to enhance neurite growth in PC12 cells, which may be related to its effects on up-regulating the expression of GAP-43 [11]. However, the medicinal formula described above is based on a rational understanding of nature, which was both utilitarian and an encyclopedia, and its effects are speculated by medical practitioners or pharmacologists. Therefore, it is important for researchers to determine the bioactive compounds from *P. cocos* that may have an effective function on learning and memory abilities in the traditional Chinese medicinal formula.

Modern pharmacological studies have shown that *P. cocos* aqueous extract can improve long-term potentiation in the hippocampus of rats and improve scopolamine-induced spatial memory impairment [12]. This study also found that pachymic acid, the medicinal component of *P. cocos*, can enhance pentobarbital-induced sleep behavior in mice through the γ-aminobutyric acid (GABAA)-ergic system [13]. Although the role of *P. cocos* in neurological diseases has gradually attracted people’s attention, it is still unclear which specific components in *P. cocos* exert their effect and the specific mechanism of the effect. In addition, previous studies have revealed that the 80% (*v*/*v*) ethanol extract of *P. cocos* induces differentiation in Neuro-2a cells [14]. However, it has not been reported whether *P. cocos* can promote the differentiation of NSPCs. Therefore, here we focused on examining the effects of *P. cocos* on NSPC differentiation and exploring the chemical basis of the promoting activity of *P. cocos* on neuronal differentiation. Ultimately, the results of this work may provide a practical foundation to guide the future discovery of new applications for neuronal differentiation-promoting activity in NSPCs.

## 2. Results

### 2.1. EEPC Induces Neuronal Differentiation and Neurite Outgrowth in Neuro-2a Cells

**Figure 1:**
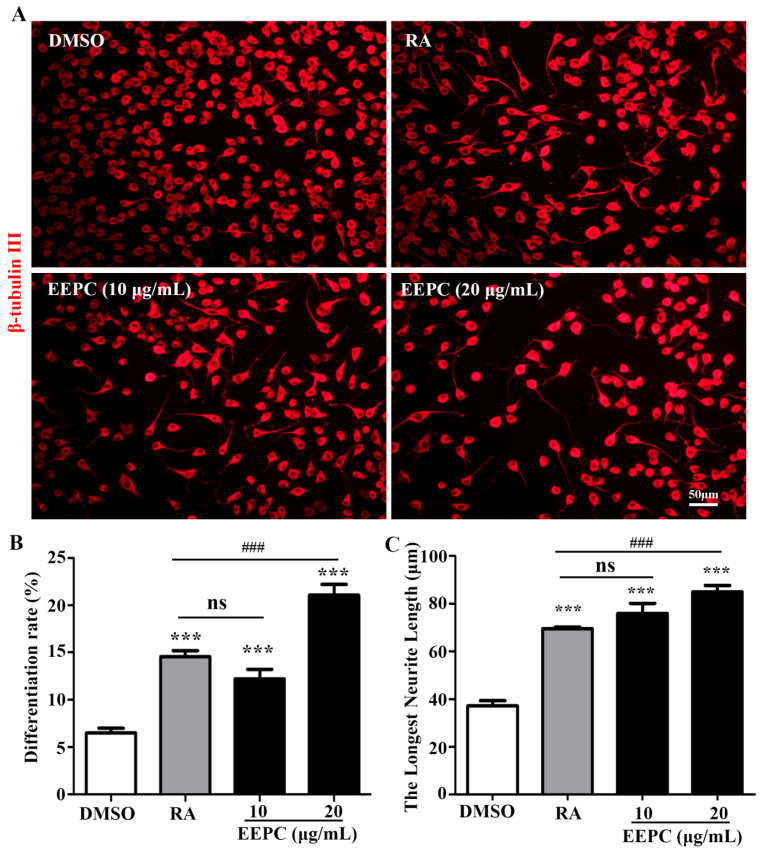
Effect of EEPC on the differentiation of Neuro-2a cells. (**A**) Neuro-2a cells were treated with EEPC at different concentrations from 1 to 20 μg/mL for 48 h. RA, retinoic acid, as a positive control. Scale bar, 50 mm. The differentiation rate (**B**) and the longest neurite length (**C**) of each differentiated cell were calculated. One-way ANOVA followed by Tukey’s test. Error bars represent SEM (*n* = 3). *** *p* < 0.001, EEPC and RA vs. DMSO; ^###^ *p* < 0.001, EEPC vs. RA; ns, no significant differences.

Our previous findings showed the ability of EEPC to induce apoptosis and differentiation in Neuro-2a neuroblastoma cells [14]. As shown in Figure 1A, untreated cells (DMSO) were round with few neurites, whereas RA-treated cells clearly displayed long neurites. Here, we compared the effects of EEPC on inducing Neuro-2a cell differentiation, including the differentiation rate (Figure 1B) and the longest neurite length (Figure 1C). EEPC showed greater activity than RA. Moreover, EEPC promoted neuronal differentiation and neurite growth of Neuro-2a cells in a concentration-dependent manner.

### 2.2. EEPC Activates JNK1/2/3 during EEPC-Induced Neuronal Differentiation

**Figure 2:**
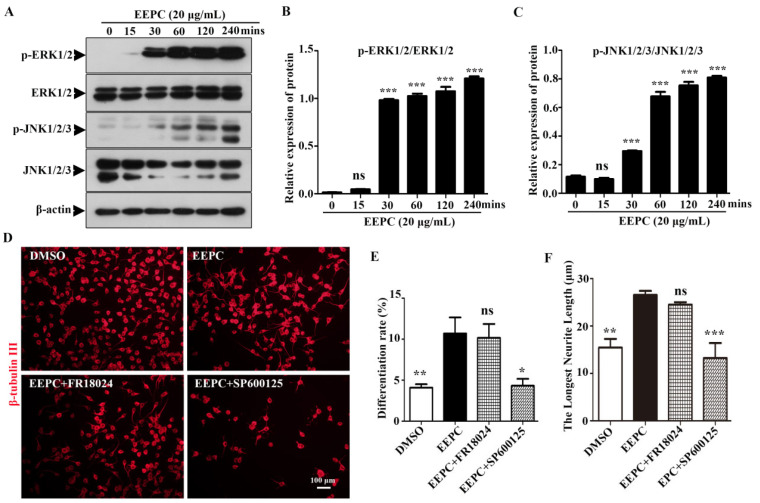
Activation of the JNK1/2/3 signaling pathway is required for EEPC induced Neuro-2a cell differentiation. (**A**–**C**) Western blot analysis was used to detect the phosphorylation (p-ERK1/2 or p-JNK1/2/3) and total (ERK1/2 or JNK1/2/3) forms of different signaling proteins. Neuro-2a cells were pretreated with different inhibitors, including ERK inhibitor (FR18024, 10 μM) or JNK inhibitor (SP600125, 10 μM) for 1 h, followed by EEPC treatment (20 μg/mL) for 48 h. Neurites were visualized by an inverted phase contrast microscope (**D**). Cell differentiation rate (**E**) and the longest length of neurites per differentiated cell (**F**) were quantified. At least 300 cells/group were analyzed in each experiment (*n* = 3), with a one-way ANOVA followed by Dunnett’s test. Error bars represent SEM (*n* = 3). ns, no significant differences; * *p* < 0.05, ** *p* < 0.01, *** *p* < 0.001, DMSO or inhibitor and EEPC cotreatment vs. EEPC single treatment.

We also tested whether the ERK1/2 and JNK1/2/3 signaling pathways were involved in the EEPC-induced neuronal differentiation by Western blot assay. As shown in Figure 2A–C, phosphorylation of ERK1/2 and JNK1/2/3 was significantly increased by EEPC treatment for 30–240 min. These results suggested that EEPC treatment promotes ERK1/2 and JNK1/2/3 phosphorylation in Neuro-2a cells, and this may be the mechanism of EEPC-induced promotion of neurite outgrowth. To further determine whether activation of ERK1/2 and JNK1/2/3 signaling is required for EEPC promoted Neuro-2a cell differentiation and neurite outgrowth, we conducted a blocking study with an ERK1/2 inhibitor (FR18024, 10 mM) and a JNK 1/2/3 inhibitor (SP600125, 10 mM). As shown in Figure 2D–F, SP600125 but not FR18024 completely block the effect of EEPC on promoting Neuro-2a cell differentiation and neurite outgrowth. These results suggest that EEPC promotes neuronal differentiation in Neruo-2a cells through activation of the JNK1/2/3 signaling pathway.

### 2.3. EEPC Promotes Neuronal Differentiation and the Morphological Maturation of Newborn Neurons in NSPCs

**Figure 3:**
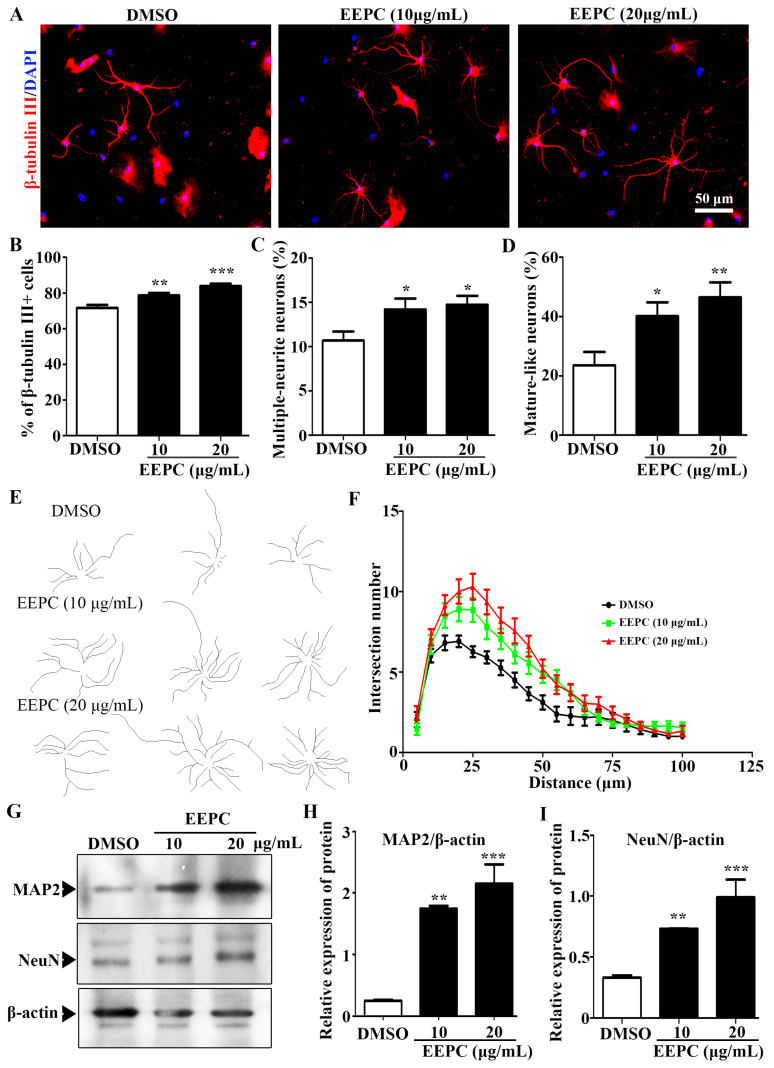
Newborn neurons derived from neural stem cells (NSCs) in the presence of EEPC developed a more mature morphology. (**A**) Immunostaining of newborn neurons for β-tubulin III (red) and nuclei with DAPI (blue), scale bar, 50 μm. The percentage of β-tubulin III positive cells (**B**), multiple-neurite neurons (**C**), and mature-like neurons (**D**) was measured. (**E**) EEPC influences mature-like neuron morphology. (**F**) The number of dendritic intersections (at 200 μm from the cell soma) was obtained by Sholl analysis. (**G**–**I**). The expression of MAP2 and NeuN was analyzed by Western blot analysis. * *p* < 0.05, ** *p* < 0.01, *** *p* < 0.001. One-way ANOVA followed by Dunnett’s test. Error bars represent SEM (*n* = 3).

We observed that the ratio of β-tubulin III positive cells (Neurons) was significantly increased from 71.6 ± 1.8% (DMSO) to 75.9 ± 1.3% (EEPC, 10 μg/mL) and 83.9 ± 1.3% (EEPC, 20 μg/mL), respectively (Figure 3A,B). This result suggested that EEPC could promote NSPC differentiation into neurons. Neuronal differentiation is a sequential, multi-step process in which cells change from progenitor cells to premature neurons and finally into mature neurons. During neuronal development, cell morphology undergoes dramatic changes, in which the neurite extension and the number of branches per neuron increase. To gain further insight into the effects of EEPC on neuronal maturation, the proportion of multi-neurite neurons (more than two branches) were measured. The results showed that the percentage of multi-neurite neurons was significantly increased from 10.68 ± 1.0% (DMSO) to 14.2 ± 1.2% (EEPC, 10 μg/mL) and 14.7 ± 1.0% (EEPC, 20 μg/mL), respectively (Figure 3C). Mature-like neurons had more and longer dendrites. It is worth noting that EEPC treatment significantly increased the percentage of mature-like neurons from 23.6 ± 4.5% (DMSO) to 40.2 ± 4.7% (EEPC, 10 μg/mL) and 46.5 ± 5.1% (EEPC, 20 μg/mL), respectively. Finally, the effect of EEPC on dentritic complexity was assessed by Sholl analysis (Figure 3E,F). Furthermore, the expression levels of neuronal markers (MAP2 and NeuN) were determined by Western blot (Figure 3G). Consistent with the results of immunofluorescence staining, the expression of MAP2 and NeuN was upregulated in EEPC-treated cells (Figure 3H,J). These results suggest that EEPC can promote the morphological maturation of newborn neurons.

### 2.4. Identification of the Chemical Constituents of EEPC Extract

**Figure 4:**
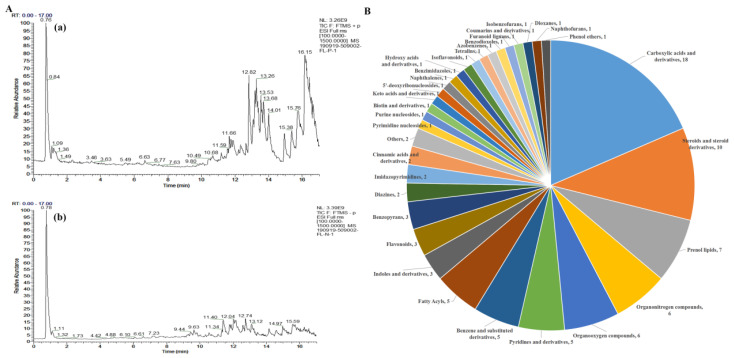
UHPLC-Q-Exactive-MS/MS analysis of the chemical constituents in the EEPC. (**A**) Positive ion mode (**a**) and Negative ion mode (**b**). (**B**) Classification of chemical constituents.

The UHPLC-Q-Exactive-MS/MS technique was chosen for our study to determine the chemical constituents of EEPC. The TIC of EEPC in positive and negative ion modes is portrayed in Figure 4A. A total of 97 compounds were identified from EEPC (Table S1), and these compounds were classified into 33 classes, including Carboxylic acids and derivatives (18.6%), Steroids and steroid derivatives (10.3%), Prenol lipids (7.2%), Organonitrogen compounds (6.2%), Organooxygen compounds (6.2%), Pyridines and derivatives (5.2%), Pyridines and derivatives (5.2%) and Benzene and substituted derivatives (5.2%) (Figure 4B). Among the compounds, the relative content of Choline (17.6%) was the highest, followed by maximum that of (3β, 5ξ, 9ξ)-3,6,19-Trihydroxyurs-12-en-28-oic acid (9.7%), Adenosine (7.2%), L-(-)-Arabitol (6.5%), and Ethyl palmitoleate (5.4%), respectively (Table 1).

### 2.5. Choline Is Identified as the Major Effective Component of EEPC

**Figure 5:**
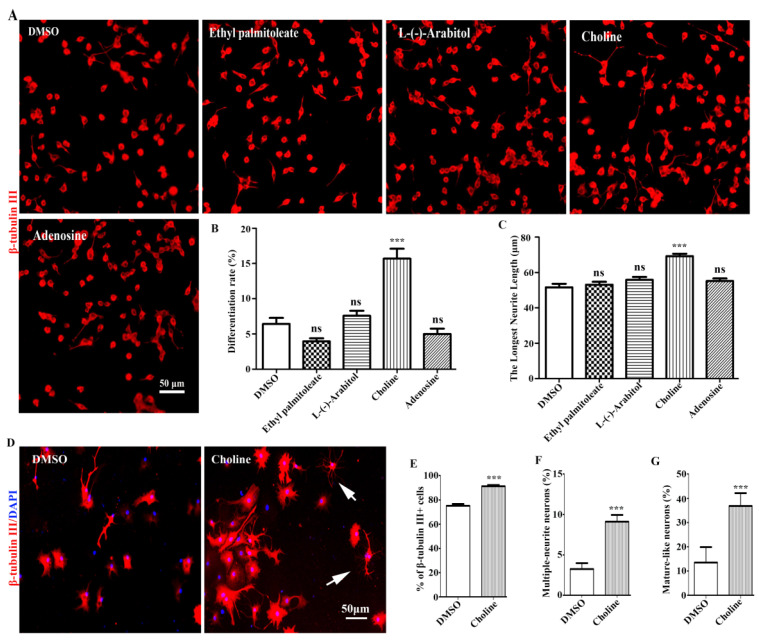
Choline was identified as the main active component of EEPC. (**A**) Neuro-2a cells were treated with Ethyl palmitoleate (3.8 μM), L-(-)-Arabitol (8.6 μM), Choline (34.4 μM), and Adenosine (5.4 μM) for 48 h. Scale bar, 50 mm. The differentiation rate (**B**) and the longest neurite length of each differentiated cell (**C**) were analyzed. (**D**) NSPCs were treated with Choline (34.4 μM), neurons were immunestained for β-tubulin III (red), and nuclei with DAPI (blue). Scale bar, 50 μm. The percentage of β-tubulin III-positive cells (**E**), multiple-neurite neurons (**F**), and mature-like neurons (**G**) was measured. ns, no significant differences, *** *p* < 0.001 compared to the DMSO.

The neuronal differentiation-promoting activities of the four compounds were first investigated using Neuro-2a cells. The results showed Adenosine, L-(-)-Arabitol, and Ethyl palmitoleate had weak effects on Neuro-2a cells differentiation; Choline had the most dramatic neuronal differentiation-promoting effects in Neuro-2a cells (Figure 5A–C). Additionally, Choline also showed obvious neuronal differentiation-promoting effects in NPCs (Figure 5D–G). Therefore, our results showed that Choline was identified as the major bioactive compound with neuronal differentiation-promoting activity in EEPC.

## 3. Discussion

The discovery of adult brain stem cells and the development of adult neurogenesis research have brought hope for the ultimate treatment of neurodegenerative diseases [15,16]. From this perspective, finding drugs that can induce the neurogenesis of adult NSPCs has become an effective therapeutic strategy for neurodegenerative diseases [17,18]. Recently, many herbal plant extracts and natural compounds were screened for promoting neurogenesis, with some of them being promising drug candidates [19,20]. In China, a number of herb ingredients known as Traditional Chinese Medicine (TCM) have a long history of use for improved learning and memory [21,22]. Therefore, these medicinal herbs provide efficient resources for drug discovery to promote neurogenesis. *P. cocos* is a well-known traditional medicinal fungus and dietary supplement. Previous studies demonstrated that *P. cocos* exhibited a series of neuropharmacological activities, including memory improvement [23], antidepressive [24] and neuroprotective [25]. Our earlier study showed the ability of EEPC to induce apoptosis and differentiation in Neuro-2a neuroblastoma cells. Results described here show that EEPC exhibited stronger activities, indicating that EEPC promoted neuronal differentiation and neurite growth of Neuro-2a cells in a concentration-dependent manner (Figure 1). However, the bioactive components and neuropharmacological mechanisms of *P. cocos* remain unclear. In this work, we explored the influence of *P. cocos* on neurogenesis and unraveled the material basis for its action.

The Neuro-2a cell line has neuron-like properties that are commonly used as a model for the study of neuronal differentiation [26]. Here we demonstrated that the 80% (*v*/*v*) ethanol extract of *P. cocos* has neuronal differentiation-promoting activity in cultured Neuro-2a cells, leading to marked neurite outgrowth. Previous studies on the extracellular signal-regulated kinase (ERK) and c-jun N-terminal (JNK) are involved in the regulation of neurite outgrowth [27]. Interestingly, although EEPC markedly activated ERK1/2 at 30 min and thereafter, inhibition of ERK activity cannot offset the promotion of EEPC on neurite outgrowth. However, inhibition of JNK activity can offset the promotion of EEPC on neurite outgrowth. These results (Figure 2) were consistent with a previous study that showed that JNK activation is required for neurite outgrowth in Neuro-2a cells. To confirm whether the effects of EEPC on neurogenesis are true, we examined the extract in primary cortical NSPC cultures. Indeed, EEPC can not only induce the differentiation of NSPCs into neuronal cell types but also promote the differentiation of newborn neurons into more mature types of cell morphology (Figure 3).

In order to obtain a better insight into the chemical constituents that could be contributing to the activity, a total of 97 metabolites were identified by UHPLC-Q-Exactive-MS/MS in EEPC (Table S1). The analysis of the relative proportion showed that there were 20 components (Table 1) accounting for 78.6% of the total amount. In addition, the neuronal differentiation-promoting activities of Choline (17.6%), Adenosine (7.2%), L-(-)-Arabitol (6.5%), and Ethyl palmitoleate (5.4%) were further confirmed both in Neuro-2a cells and NSPCs. Choline, which accounts for the largest proportion of EEPC, was proven to have significant neuronal differentiation-promoting activity. Regarding possible active ingredients, choline is a precursor to many important compounds and is recognized as an essential nutrient [28]. Existing evidence suggests that choline plays an important role in neural tube closure and brain development during the perinatal period [29,30]. Moreover, dietary choline intake affects the structure and function of hippocampal pyramidal cells [31]. Notably, these structural changes are often associated with memory function. Therefore, studies have also found that perinatal choline supplementation in rodents can enhance memory and learning function, with effects lasting throughout life [32]. On the contrary, choline deficiency during these sensitive periods results in the persistence of memory and cognitive deficits [33]. In addition, it was also discovered that maternal choline supplementation significantly improves spatial learning and induces adult hippocampal neurogenesis in a Down syndrome mouse model [34]. Although the cellular mechanisms by which choline exerts these effects are unclear, numerous studies have implicated choline in the regulation of stem cell proliferation and differentiation through DNA methylation and altered gene expression [35,36]. We also found choline to have dramatic neuronal differentiation-promoting effects in Neuro-2a cells (Figure 5A–C) and obvious neuronal differentiation-promoting effects in NSPCs (Figure 5D–G). Therefore, choline was considered to be the main active component of *P. cocos* that promotes the neuronal differentiation of NSPCs.

## 4. Materials and Methods

### 4.1. Reagents

**Figure 6:**
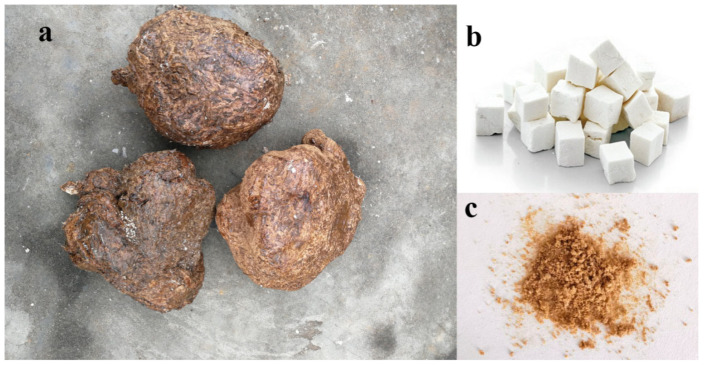
*Poria cocos* (Schw.) Wolf (**a**). The dried sclerotia of *Poria cocos* (Schw.) Wolf (**b**). The 80% (*v*/*v*) ethanol extract of *Poria cocos* (Schw.) Wolf (**c**).

*P. cocos* (Figure 6a) was purchased from Hunan Province, China; FR180204, SP600125, Primary antibodies against anti-ERK1/2, anti-p-ERK1/2, anti-JNK1/2/3, anti-p-JNK1/2/3, anti-MAP2, anti-NeuN, anti-β-actin, HRP-conjugated anti-mouse, and anti-rabbit were purchased from Beyotime Biotechnology (Shanghai, China); anti-β-tubulin III, DMSO, and MTT were purchased from Sigma (St. Louis, MO, USA). Minimum Eagle’s Medium (MEM), foetal bovine serum (FBS), penicillin, and streptomycin were obtained from Hyclone (Logan, UT, USA). Choline, Adenosine, L-(-)-Arabitol, and Ethyl palmitoleate are from Yuanye Biotechnology (Shanghai, China); all other chemicals and reagents are of analytical grade.

### 4.2. Preparation of 80% (v/v) Ethanol Extracts of P. cocos (EEPC)

The EEPC was prepared as previously described [37]. Dried *P. cocos* sclerotia (Figure 6b) were ground to powder and extracted twice with 10 volumes of 80% ethanol in a reflux condenser at 85–90 °C for 3 h. After filtration through a 0.2 μm filter, the extract was concentrated and vacuum evaporated to undergo lyophilization. The extract in solid form (Figure 6c) was dissolved in DMSO before the experiment.

### 4.3. Identification and Analysis of Chemical Constituents in EEPC Using UHPLC-Q-Exactive-MS/MS

Chromatographic analysis was performed using a Thermo Vanquis UHPLC system (Thermo Fisher Scientific, Waltham, MA, USA). The chromatographic separation was carried out on an Agilent Zorbax Eclipse C18 (1.8 μm × 2.1 × 100 mm) chromatographic column using gradient elution. The optimal mobile phase was 0.1% formic acid aqueous solution (solvent A) and acetonitrile (solvent B); the column temperature was maintained at 35 °C, and the sample chamber temperature was set to 8 °C. Gradient elution settings were: 0–5 min, 2% B; 5–20 min, 2%–98% B; 20–25 min, 98%–2% B; 25–30 min, 2% B. The flow rate was 0.30 mL·min^−1^, and the injection volume was 2 µL.

The mass spectrometer with a heated electrospray ionization source was operated in positive and negative ion modes. The key parameters: spray voltage of +3.8 kV/−3.2 kV; 45 arbitrary units (Arbs) sheath gas flow; 15 Arbs auxiliary gas flow; 1 Arbs purge gas flow; capillary temperature of 350 °C; auxiliary gas heater temperature of 300 °C were applied. Scan modes: full MS at 70,000 FWHM resolution and data-dependent MS/MS at 17,500 FWHM resolution; stepped normalized collision energies ranged at 20, 40, and 60 eV, while the scan range was *m*/*z* 75–1050. Data were performed using Compound Discoverer 3.0, Thermo mzCloud, and Thermo mzValut 2.3 software (Thermo Scientific, Fremont, CA, USA), respectively.

### 4.4. Cell Culture

Mouse neuroblastoma Neuro-2a cells were gifted by Dr. Gen-yun Tang from Hunan University of Medicine, China. Cells grown in MEM medium (Hyclone) supplemented with 10% heat-inactivated fetal bovine serum and 1% penicillin/streptomycin were maintained at 37 °C in a 5% CO_2_ humidified atmosphere. Cells were passaged every 3–4 days.

Primary NSPCs were isolated and cultured as previously described [38]. For in vitro differentiation, single cells dissociated from neurospheres were seeded at a density of 2 × 10^4^ cells/mL on coverslips coated with poly-D-lysine (100 ng/mL) and laminin (20 μg/mL). NSPCs were maintained in differentiation medium (DMEM/F12 supplemented with 10% FBS and 1% penicillin-streptomycin) for 5 days.

### 4.5. Cytotoxicity Test

The in vitro cytotoxicity of EEPC was evaluated by an MTT assay. For the assay, 5 × 10^3^cells/well were grown overnight in 96-well microtiter plates. Afterwards, various concentrations of EEPC (5, 10, 20, 50, and 100 µg/mL) were added. After 24 h of incubation, the media containing EEPC were carefully removed, and MTT solution (0.5 mg/mL in MEM) was added to each well and further incubated for 4 h. Formazon crystals were dissolved in 200 µL of DMSO, and the absorbance was measured by a microplate reader at 570 nm.

### 4.6. Western Blotting

Cells were washed in ice-cold PBS and lysed in RIPA buffer (mixed with protease/phosphatase inhibitors). The protein concentration was quantified using a BCA protein assay kit. Total cell lysates were boiled for 10 min, then separated by SDS-PAGE and transferred to polyvinylidene fluoride (PVDF) membranes. The membranes were probed with phosphorylated and total ERK 1/2 and JNK1/2/3 antibodies and then subsequently with secondary antibodies, followed by electrochemiluminescence (ECL) detection.

### 4.7. Differentiation Assay

Neuro-2a cells were seeded at a density of 1 × 10^4^ cells/mL into 24-well plates and maintained at 37 °C under a 5% atmosphere for 24 h. After 24 h of incubation, the culture medium was changed to differentiation medium (MEM supplied with 0.5% FBS and 1% penicillin/streptomycin) in the presence of EEPC (10 and 20 μg/mL) and Retinoic acid (RA, 10 μM) for 48 h. After 48 h of differentiation, cells were labeled by immunostaining with an antibody against β-tubulin III to show the presence of neurite. Images were taken under a phase contrast microscope. A protrusion with a length greater than the diameter of the cell body is defined as a neurite. The differentiation rate and the longest neurite length of each differentiated cell were measured by Image J software (Version 1.8.0).

### 4.8. Immunostaining

Cells were washed using PBS and fixed in 4% paraformaldehyde (PFA) for 20 min. Fixed cells were permeabilized in PBS with 0.4% Triton X-100 for 30 min and blocked for 20 min at room temperature in blocking buffer (PBS with 5% goat serum and 1% bovine serum albumin). The cells were incubated at 4 °C overnight with mouse β-tubulin III antibody (1:1000, *v*/*v*), followed by incubation with Alexa Fluor-546 goat anti-mouse IgG secondary antibodies (1:2000, *v*/*v*) for 1 h at room temperature. DAPI was added to visualize the nuclei. Images were captured using a fluorescence microscope (Olympus IX71, Tokyo, Japan).

### 4.9. Statistical Analysis

The results are expressed as the mean ± standard error of the mean (SEM). Significant differences between the two groups were evaluated using a Student’s *t* test and a one-way ANOVA test to assess the differences between the relevant control and each experimental group. A value of *p* < 0.05 was considered statistically significant.

## 5. Conclusions

In this study, we showed that EEPC treatment promotes neurogenesis in NSPCs in vitro. Based on these results, it is suggested that *P. cocos* has a positive effect on learning and memory functions, and this might be due to its neurogenesis effect. In addition, whether *P. cocos* can still promote endogenous neurogenesis and improve learning and memory in vivo still needs to be further verified by animal experiments. Meanwhile, in-depth mechanisms are required for more concrete information on the pharmacological features of *P. cocos*. Nevertheless, to the best of our knowledge, this is the first investigation of the neurogenesis effect of *P. cocos*, and the main biologically active substance is choline. Our findings provide an experimental basis for the application of *P. cocos* in the treatment of neurodegenerative diseases.

## Figures and Tables

**Table 1:** Identification of chemical constituents of EEPC by UHPLC-MS/MS (Relative proportion ≥ 1%).

NO.	Name	Formula	Class	M.W.	RT [min]	Relative Proportion (1%)
1	Choline	C_5_ H_13_NO	Organonitrogen compounds	103.09982	0.769	17.75
2	L-Glutamic acid	C_5_H_9_NO_4_	Carboxylic acids and derivatives	147.05274	0.79	1.14
3	N,N-Diethylethanolamine	C_6_H_15_NO	Organonitrogen compounds	117.11529	0.797	2.20
4	Betaine	C_5_H_11_NO_2_	Carboxylic acids and derivatives	117.0789	0.809	1.82
5	Trigonelline	C_7_H_7_NO_2_	Pyridines and derivatives	137.04733	0.813	2.46
6	L-(-)-Arabitol	C_5_H_12_O_5_	Organooxygen compounds	152.06812	0.82	6.51
7	Cytosine	C_4_H_5_N_3_O	Diazines	111.0433	0.837	1.81
8	Cytidine	C_9_H_13_N_3_O_5_	Pyrimidine nucleosides	243.0849	0.84	1.01
9	Adenosine	C_10_H_13_ N_5_O_4_	Purine nucleosides	267.09604	1.243	7.23
10	Acetophenone	C_8_H_8_O	Organooxygen compounds	120.05739	1.321	1.14
11	Leucine	C_6_H_13_NO_2_	Carboxylic acids and derivatives	131.0944	1.36	2.53
12	L-Phenylalanine	C_9_H_11_NO_2_	Carboxylic acids and derivatives	165.07866	2.292	1.50
13	Phenylacetylene	C_8_H_6_	Benzene and substituted derivatives	102.0469	2.292	1.46
14	Picolinic acid	C_6_H_5_NO_2_	Pyridines and derivatives	123.03191	3.231	1.02
15	Triphenylphosphine oxide	C_18_H_15_OP	Benzene and substituted derivatives	278.0851	10.705	3.17
16	2-Amino-1,3,4-octadecanetriol	C_18_H_39_NO_3_	Organonitrogen compounds	317.29191	11.185	3.17
17	(3β,5ξ,9ξ)-3,6,19-Trihydroxyurs-12-en-28-oic acid	C_30_H_48_O_5_	Prenol lipids	488.34864	11.697	9.67
18	Bis(4-ethylbenzylidene)sorbitol	C_24_H_30_O_6_	Dioxanes	414.20291	11.918	2.50
19	18-β-Glycyrrhetinic acid	C_30_H_46_O_4_	Prenol lipids	470.33821	13.044	1.05
20	Ethyl palmitoleate	C_18_H_34_O_2_	Fatty Acyls	282.25502	14.152	5.42
21	3-Acetyl-11-keto-β-boswellic acid	C_32_H_48_O_5_	Prenol lipids	512.34893	14.93	2.90
22	4-Methoxycinnamic acid	C_10_H_10_O_3_	Cinnamic acids and derivatives	178.06257	16.726	1.20

## Data Availability

Not applicable.

## Supplementary Materials

The following supporting information can be downloaded at: https://www.mdpi.com/article/10.3390/molecules28227480/s1, Table S1: Identification of chemical constituents of EEPC by UHPLC-Q-Exactive—MS/MS.

## References

[1] Innocenti G.M. (2022). Defining neuroplasticity. Handb. Clin. Neurol..

[2] Mehler M.F., Gokhan S. (2001). Developmental mechanisms in the pathogenesis of neurodegenerative diseases. Prog. Neurobiol..

[3] Zhou Z., Shi B., Xu Y., Zhang J., Liu X., Zhou X., Feng B., Ma J., Cui H. (2023). Neural stem/progenitor cell therapy for Alzheimer disease in preclinical rodent models: A systematic review and meta-analysis. Stem Cell Res. Ther..

[4] Zhao L., Liu J.W., Shi H.Y., Ma Y.M. (2021). Neural stem cell therapy for brain disease. World J. Stem Cells.

[5] Yang Y., Huang X.L., Jiang Z.M., Li X.F., Qi Y., Yu J., Yang X.X., Zhang M. (2022). Quantification of Chemical Groups and Quantitative HPLC Fingerprint of *Poria cocos* (Schw.) Wolf. Molecules.

[6] Wang W., Dong H., Yan R., Li H., Li P., Chen P., Yang B., Wang Z. (2015). Comparative study of lanostane-type triterpene acids in different parts of *Poria cocos* (Schw.) Wolf by UHPLC-Fourier transform MS and UHPLC-triple quadruple MS. J. Pharm. Biomed. Anal..

[7] Song Z., Bi K., Luo X., Chan K. (2002). The isolation, identification and determination of dehydrotumulosic acid in *Poria cocos*. Anal. Sci..

[8] Li H., Li S.L., Gong L., Wang J.L., Li Y.Z., Wu Z.H. (2008). The effects of an herbal medicine Bu-Wang-San on learning and memory of ovariectomized female rat. J. Ethnopharmacol..

[9] Zhu Y., Duan X., Cheng X., Cheng X., Li X., Zhang L., Liu P., Su S., Duan J.A., Dong T.T. (2016). Kai-Xin-San, a standardized traditional Chinese medicine formula, up-regulates the expressions of synaptic proteins on hippocampus of chronic mild stress induced depressive rats and primary cultured rat hippocampal neuron. J. Ethnopharmacol..

[10] Liu J.P., Feng L., Zhang M.H., Ma D.Y., Wang S.Y., Gu J., Fu Q., Qu R., Ma S.P. (2013). Neuroprotective effect of Liuwei Dihuang decoction on cognition deficits of diabetic encephalopathy in streptozotocin-induced diabetic rat. J. Ethnopharmacol..

[11] Zhang Q., Zhang Z.J., Wang X.H., Ma J., Song Y.H., Liang M., Lin S.X., Zhao J., Zhang A.Z., Li F. (2016). The prescriptions from Shenghui soup enhanced neurite growth and GAP-43 expression level in PC12 cells. BMC Complement. Altern. Med..

[12] Smriga M., Saito H., Nishiyama N. (1995). Hoelen (*Poria Cocos* Wolf) and ginseng (*Panax Ginseng* C. A. Meyer), the ingredients of a Chinese prescription DX-9386, individually promote hippocampal long-term potentiation in vivo. Biol. Pharm. Bull..

[13] Shah V.K., Choi J.J., Han J.Y., Lee M.K., Hong J.T., Oh K.W. (2014). Pachymic Acid Enhances Pentobarbital-Induced Sleeping Behaviors via GABAA-ergic Systems in Mice. Biomol. Ther..

[14] Xia Jiang J.X. (2022). Zhaotun Hu, Xiaoliang Xiang, Ethanol extract of *Poria cocos* induces apoptosis and differentiation in Neuro-2a neuroblastoma cells. Bangladesh J. Pharmacol..

[15] Stepien T. (2021). Neurogenesis in neurodegenerative diseases in the adult human brain. Postep. Psychiatr. Neurol..

[16] Peretto P., Bonfanti L. (2015). Adult neurogenesis 20 years later: Physiological function vs. brain repair. Front. Neurosci..

[17] Chang K.A., Kim J.A., Kim S., Joo Y., Shin K.Y., Kim S., Kim H.S., Suh Y.H. (2012). Therapeutic potentials of neural stem cells treated with fluoxetine in Alzheimer’s disease. Neurochem. Int..

[18] Grochowski C., Radzikowska E., Maciejewski R. (2018). Neural stem cell therapy-Brief review. Clin. Neurol. Neurosurg..

[19] Deb S., Phukan B.C., Dutta A., Paul R., Bhattacharya P., Manivasagam T., Thenmozhi A.J., Babu C.S., Essa M.M., Borah A. (2020). Natural Products and Their Therapeutic Effect on Autism Spectrum Disorder. Adv. Neurobiol..

[20] Park H.R., Kim J.Y., Lee Y., Chun H.J., Choi Y.W., Shin H.K., Choi B.T., Kim C.M., Lee J. (2016). PMC-12, a traditional herbal medicine, enhances learning memory and hippocampal neurogenesis in mice. Neurosci. Lett..

[21] Ren H., Gao S., Wang S., Wang J., Cheng Y., Wang Y., Wang Y. (2022). Effects of Dangshen Yuanzhi Powder on learning ability and gut microflora in rats with memory disorder. J. Ethnopharmacol..

[22] Sun C., Liu J., Li N., Liu M., Luo Z., Li H. (2021). Traditional Chinese Medicine Shenmayizhi Decoction Ameliorates Memory and Cognitive Impairment Induced by Multiple Cerebral Infarctions. Evid.-Based Complement. Altern. Med. eCAM.

[23] Wu K.J., Chen Y.F., Tsai H.Y., Wu C.R., Wood W.G. (2012). Guizhi-Fuling-Wan, a Traditional Chinese Herbal Medicine, Ameliorates Memory Deficits and Neuronal Apoptosis in the Streptozotocin-Induced Hyperglycemic Rodents via the Decrease of Bax/Bcl2 Ratio and Caspase-3 Expression. Evid.-Based Complement. Altern. Med. eCAM.

[24] Zhang W., Chen L., Li P., Zhao J., Duan J. (2018). Antidepressant and immunosuppressive activities of two polysaccharides from *Poria cocos* (Schw.) Wolf. Int. J. Biol. Macromol..

[25] Lv Q., Di X., Bian B., Li K., Guo J. (2022). Neuroprotective Effects of *Poria cocos* (Agaricomycetes) Essential Oil on Abeta1-40-Induced Learning and Memory Deficit in Rats. Int. J. Med. Mushrooms.

[26] Jiang X., Tang G., Yang J., Ding J., Lin H., Xiang X. (2020). Synthesis of some new acylhydrazone compounds containing the 1,2,4-triazole structure and their neuritogenic activities in Neuro-2a cells. RSC Adv..

[27] Waetzig V., Herdegen T. (2003). The concerted signaling of ERK1/2 and JNKs is essential for PC12 cell neuritogenesis and converges at the level of target proteins. Mol. Cell. Neurosci..

[28] Hollenbeck C.B. (2012). An introduction to the nutrition and metabolism of choline. Cent. Nerv. Syst. Agents Med. Chem..

[29] Derbyshire E., Obeid R. (2020). Choline, Neurological Development and Brain Function: A Systematic Review Focusing on the First 1000 Days. Nutrients.

[30] Caudill M.A. (2010). Pre- and postnatal health: Evidence of increased choline needs. J. Am. Diet. Assoc..

[31] Bastian T.W., von Hohenberg W.C., Kaus O.R., Lanier L.M., Georgieff M.K. (2022). Choline Supplementation Partially Restores Dendrite Structural Complexity in Developing Iron-Deficient Mouse Hippocampal Neurons. J. Nutr..

[32] Tees R.C. (1999). The influences of rearing environment and neonatal choline dietary supplementation on spatial learning and memory in adult rats. Behav. Brain Res..

[33] Mudd A.T., Getty C.M., Sutton B.P., Dilger R.N. (2016). Perinatal choline deficiency delays brain development and alters metabolite concentrations in the young pig. Nutr. Neurosci..

[34] Velazquez R., Ash J.A., Powers B.E., Kelley C.M., Strawderman M., Luscher Z.I., Ginsberg S.D., Mufson E.J., Strupp B.J. (2013). Maternal choline supplementation improves spatial learning and adult hippocampal neurogenesis in the Ts65Dn mouse model of Down syndrome. Neurobiol. Dis..

[35] Blusztajn J.K., Mellott T.J. (2012). Choline nutrition programs brain development via DNA and histone methylation. Cent. Nerv. Syst. Agents Med. Chem..

[36] Fujita Y., Nagakura T., Uchino H., Inazu M., Yamanaka T. (2021). Functional Expression of Choline Transporters in Human Neural Stem Cells and Its Link to Cell Proliferation, Cell Viability, and Neurite Outgrowth. Cells.

[37] Jeong J.W., Lee H., Han M., Kim G.Y., Hong S., Park C., Choi Y. (2014). Ethanol extract of *Poria cocos* reduces the production of inflammatory mediators by suppressing the NF-kappaB signaling pathway in lipopolysaccharide-stimulated RAW 264.7 macrophages. BMC Complement. Altern. Med..

[38] Xiang X., Zhuang X., Li S., Shi L. (2017). Arhgef1 is expressed in cortical neural progenitor cells and regulates neurite outgrowth of newly differentiated neurons. Neurosci. Lett..

